# Small-scaled association between ambient temperature and campylobacteriosis incidence in Germany

**DOI:** 10.1038/s41598-020-73865-9

**Published:** 2020-10-14

**Authors:** Julia Oberheim, Christoph Höser, Guido Lüchters, Thomas Kistemann

**Affiliations:** 1 Institute for Hygiene and Public Health, University Hospital Bonn, GeoHealth Centre, Venusberg-Campus 1, 53127 Bonn, Germany; 2 Center for Development Research (ZEF), University of Bonn, Genscherallee 3, 53113 Bonn, Germany

**Keywords:** Epidemiology, Gastrointestinal diseases, Infectious diseases, Climate change, Bacteriology, Environmental microbiology

## Abstract

Campylobacteriosis is the leading bacterial cause of human diarrheal illness worldwide. Campylobacteriosis incidence exhibits seasonality and has been attributed to ambient temperature. However, the role of ambient temperature on campylobacteriosis remains poorly understood. To examine the impact of ambient temperature on local campylobacteriosis in Germany, weekly incidences on NUTS-3 level were analysed using a novel small-scaled approach, regression and time lags. Campylobacteriosis incidence correlated positively with temperatures between − 5 and 28 °C. The sigmoid regression model estimated an incidence increase of 0.52 per 5 °C temperature rise in the observation period. The weekly average of daily minimum temperature was most significant at a time lag of two weeks and showed the steepest incidence increase of 0.13 per 1 °C temperature increase in a temperature corridor of 5.1 to 12.2 °C. The impact of average minimum temperatures on campylobacteriosis incidence is crucial, likely to be indirect and especially relevant in the recent part of the infection chain. Vectors or human behaviour are presumably more directly linked with temperature than the pathogen’s microbiology and should be examined. These variables outweigh the direct temperature-pathogen relationship when the whole chain of infection is considered. In the context of climate change, campylobacteriosis is likely to increase in Germany due to an increased temperature effect.

## Introduction

*Campylobacter* spp. is one of the leading bacterial causes for human gastroenteritis worldwide^1^. In Germany, campylobacteriosis represents the most common cause of bacterial foodborne illness, and cases show a continuous upward trend from 2005 onwards^2,3^. With 67,872 cases in 2018, Germany has now become the EU country with the highest case numbers^3^. Among several known pathogenic subspecies, *Campylobacter jejuni* and *Campylobacter coli* are the most commonly associated subspecies for human campylobacteriosis^2^. The transmission route of *Campylobacter* spp. is mainly believed to be foodborne, via the consumption of undercooked meat and meat products, as well as unpasteurised milk or contaminated drinking water, fruit and vegetables. A proportion of cases occur following contact with domestic animals, working or living on a farm, travelling or frequent eating out at restaurants^4–6^. Further potential sources of infection identified include interacting with water bodies, such as rivers and surface waters during recreational activities^7^. Vectors, such as flies or litter beetles have been discussed as transmission vehicles on farms as well as at food preparation level^2,8,9^. The relative proportion of each of the named sources and transmission routes to the total burden of disease is unknown. However, the consumption of contaminated poultry is believed to be a major transmission route^2,8^. Contamination of broiler flocks mainly occurs through contact with faeces during slaughter, resulting in 30 to 54% of all carcasses in Germany found to be *Campylobacter*-positive^9^. The success of *Campylobacter* spp. transmission is closely related to its bacterial fitness. *Campylobacter* spp. is thermophilic and microaerophilic, replicating at an optimum growth temperature of 42 °C and in an environment of 5% of oxygen^10^. These conditions have been interpreted as an adaptation to its major habitat, the gastrointestinal tracts of warm-blooded avians^10^. Due to its thermophilic character replication outside the host as well as at ambient temperatures is unlikely^10^. Despite this, *Campylobacter* spp. can survive over a wide range of temperatures and climatic conditions for an extended period. Its genotype determines campylobacters’ fitness in the host and a range of environmental conditions^11^. For protection against adverse atmospheric conditions and to create a microaerophilic habitat, *Campylobacter* spp. forms biofilms and a variety of antioxidant enzymes, such as superoxide dismutase (SOD) and catalase (KatA)^12–16^. As a response to heat stress, *Campylobacter* spp. produces several heat shock proteins, which could explain its high optimal growth temperature^17^. Under temperature stress *Campylobacter* spp. has the strain-specific ability to transform from a spiral shape into a coccoid form, although it remains controversial if this state is able to increase its resistance^18–20^. Furthermore, cocci cell membrane formed at 4 °C resemble those of spirals which have been associated with higher viability^20^. At freezing temperatures of − 20 °C, *C. jejuni* can survive two to five months. In wet and cold environmental conditions of around 5 °C, such as in aquatic niches survival is especially prolonged for several weeks up to four months^21,22^. In contrast, with increasing temperature, inactivation of campylobacters accelerates, and survival at room temperature of 20 °C is only restricted to a few days^19,22^.

In Germany, campylobacteriosis incidence has been found to underly a seasonal pattern peaking in the summer months and again in early January. In other temperate climates, including Great Britain, Denmark, Sweden, Austria, and New Zealand a similar seasonality has been observed^5,23–25^. The seasonality of campylobacteriosis infection and its association with temperature is well documented^23,26,27^. Tam et al. found a linear relationship with a 5% increase in cases per 1 °C rise of mean weekly temperature in England and Wales^28^. Djennad et al. provided a strong association of campylobacteriosis and temperature on a high-resolution for the UK with limitations in the data background^29^. No association between temperature and campylobacteriosis was found by Lal et al. for New Zealand^30^. In Germany, the relationship between temperature and human campylobacteriosis has previously been studied on a low resolution and for selected regions^27,31^. However, the exact mechanisms of the seasonality remain unresolved to-date. To reveal the mechanisms underlying campylobacteriosis seasonality, this study aimed at studying campylobacteriosis incidence and temperature parameters using a high temporal and spatial resolution for the whole of Germany over an observation period of 14 years.

If the temperature is the primary driver of campylobacteriosis outcome and causes its seasonality, the association should be reproducible on a small-scaled resolution. The results would add evidence to the influence of temperature on campylobacteriosis incidence.

## Methods

### Epidemiological data

Campylobacteriosis is a mandatory reportable disease in Germany. Weekly cases and incidence data (cases per 100,000 inhabitants) were obtained from the reports of the Robert Koch Institute (RKI) from 2001 to 2014, covering 730 weeks of reporting including weeks with zero cases^32^. The case week is defined as the week a case is reported, which is the closest date to symptom onset available. The RKI counts four conditions as cases: (a) Clinical epidemiological cases, (b) clinical laboratory-diagnosed cases, (c) laboratory-diagnosed cases with unfulfilled clinical presentation, and (d) laboratory-diagnosed cases with unknown clinical presentation. Campylobacteriosis data covers the whole of Germany, and the maximal regional resolution on NUTS-3 level has been examined. NUTS-3 regions represent 413 reporting areas (‘Kreise’, ‘kreisfreie Städte’) which have a population of 33,807 to 1,798,836 inhabitants. Data from 2001 onwards have been selected due to a newly established reporting system in 2001, according to the German Infection Protection Act. Thus, the standards of detection and reporting remained constant during the observation period.

### Weather data

Temperature data from the year 2000 to 2014 with a daily resolution (5,479 days in total) was obtained from the European Climate Assessment & Dataset project (Version 04/11/2015), a project initiated by the European Climate Support Network of GIE_EUMETNET^33^. The temporal coverage of meteorological data contains an additional year compared to the epidemiological data in order to relate precedent temperature parameters to incidences that occur with a specified time lag. Raw data contained the daily minimum, mean and maximum temperature. The applied data version provides a 0.25 * 0.25-degree regular grid resolution and covers the whole of Europe. The file had to be reduced to the study area of Germany, with a coverage spanning from 5–16° E longitude to 47–56° N latitude using Open GrADS (Version 2.0).

### GIS data

GIS shapefiles were used to provide spatial information for reporting area-based incidence values. The GIS data contained reporting areas as well as NUTS-3 level IDs and was obtained from the German Federal Agency of Cartography and Geodesy (BKG)^34^. The BKG data set was enhanced for the Berlin area due to a higher spatial resolution of the RKI incidence dataset in this region. The senatorial administration of Berlin provided more detailed data for the reporting areas of Berlin^35^. Both shapefiles were intersected using ArcGIS for Desktop (Version 10.4.1) to provide a complete map congruent with RKI data.

### Data processing

**Figure 1:**
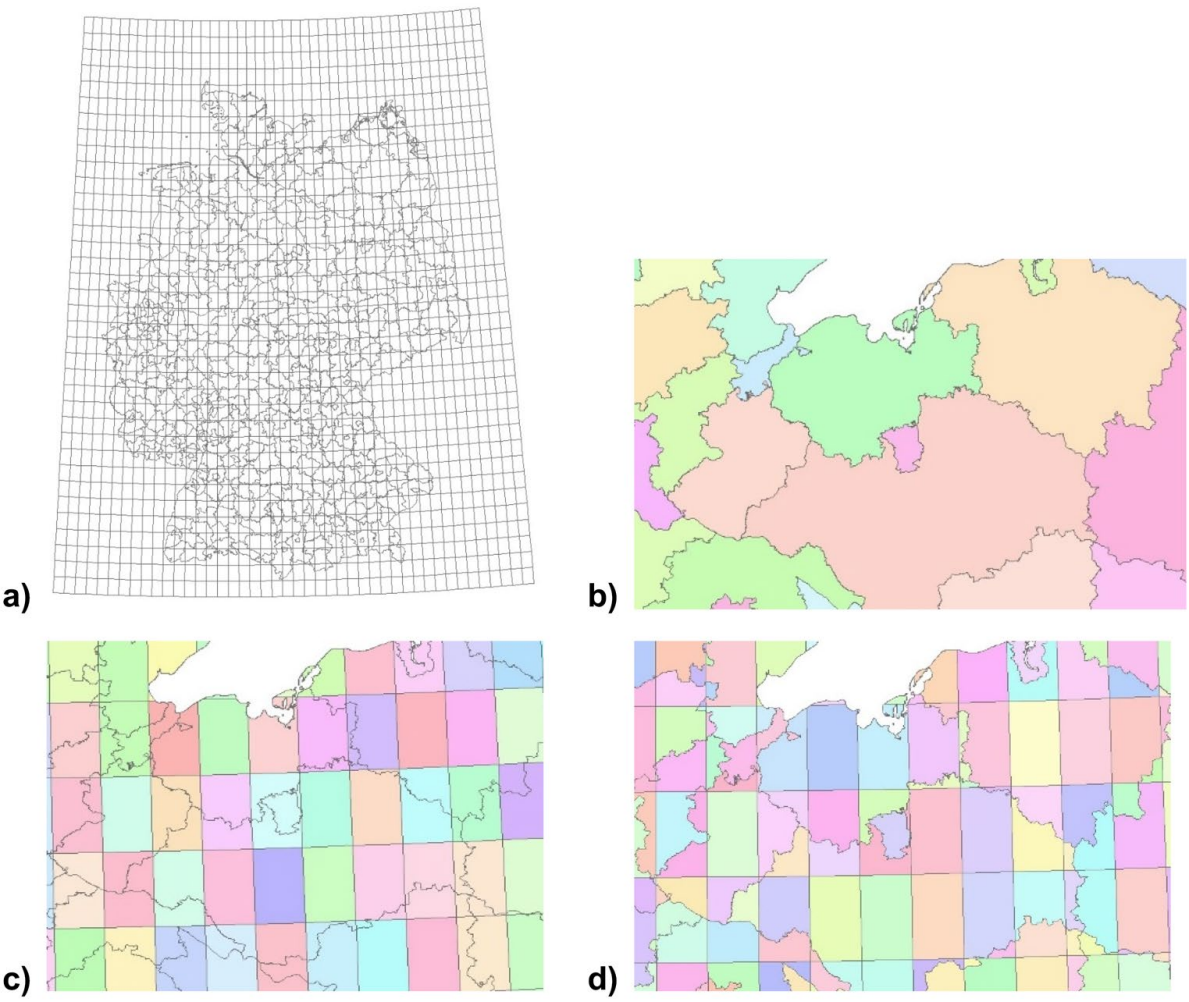
GIS data processing: assigning gridded weather data to reporting areas. (**a**) gives an overview of the gridded weather cells, overlapped with incidence reporting areas of Germany. The figures have been created using Spatial Analyst tools, as implemented in ArcGIS for Desktop (Version 10.4.1, URL: https://desktop.arcgis.com/de/arcmap/10.4/get-started/setup/arcgis-desktop-quick-start-guide.htm).

A harmonisation of weather and incidence data was carried out to obtain spatial and temporal data compatibility. Figure 1a gives an overview of the gridded weather cells, overlapped with incidence reporting areas of Germany. The 0.25-degree grid resolution of weather data was recalculated to the reported incidences on NUTS-3 level using GIS data management tools. To the reporting-area shape of incidences (Fig. 1b), a fishnet congruent to the weather raster layer (Fig. 1c) was added. Subsequently, the created fishnet was intersected with the shapefile containing the reporting areas (Fig. 1d). Every subarea of a reporting area was weighted according to its proportional size of the total respective reporting area to calculate a total reporting area weather. This proportion was used to calculate a total reporting area weather for each reporting area.

Temporal harmonisation of daily resolved temperature data and weekly reported incidence data was carried out by aggregating temperature data to a weekly resolution using HeidiSQL (Version 9.4.0.5125). To avoid the loss of extreme values, the weekly maximum, average, and minimum values of each temperature parameter and each reporting week were stored. An SQL routine sorted all resulting 301,490 reports (730 reporting weeks in the observation period, 413 reporting areas in Germany) by temperature conditions in ascending order, for each temperature parameter separately. To solve the problem of unequal population densities among the reporting areas, classes of 5,000 reports were created, cases and population summed up for all 5,000 reports to calculate a single incidence for each class. For each class, the temperature median of this class has been calculated. A class size of 5,000 reports was chosen to provide a robust statistical background. Note that every class is heterogeneous regarding temporal and spatial linkage, so one class contains reports from different reporting areas and reporting weeks. Moreover, time lags (named lagtypes in the following) were established between each weekly median class temperature and calculated class incidence ranging between a 1-week up to an eight-week time lag. The shortest time lag, lagtype 1, compares the incidence reporting week with the temperature conditions of the preceding week and lagtype 2 compares the incidence reporting week with the temperature conditions from two weeks ago for example.

**Figure 2:**
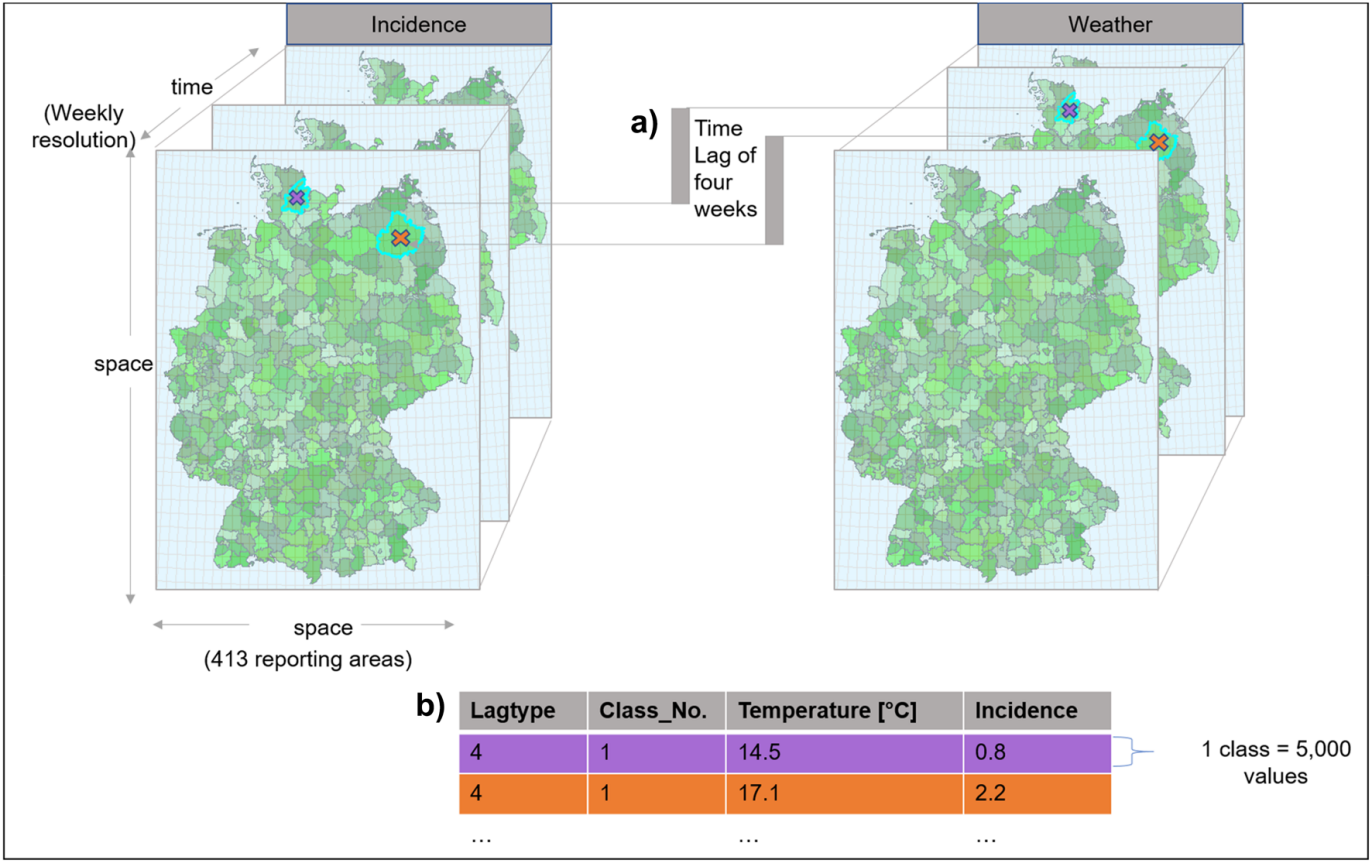
Explaining the study design: E.g. for lagtype 4, the weather week four weeks prior to the incidence week has been examined (**a**). Temperature parameter values were sorted in ascending order and aggregated into classes containing 5,000 reports. For each class, a single incidence was calculated, adding up cases and population for all 5,000 reports to create a single class incidence and a median class temperature (**b**). This diagram was created using Microsoft Office 365 ProPlus (Version 2001, URL: https://www.microsoft.com/de-de/microsoft-365). The inserted maps have been created using Spatial Analyst tools, as implemented in ArcGIS for Desktop (Version 10.4.1, URL: https://desktop.arcgis.com/de/arcmap/10.4/get-started/setup/arcgis-desktop-quick-start-guide.htm).

Data processing resulted in a table for statistical analysis which contained the median of nine different temperature parameters (maximum, minimum, and average of each temperature parameter). For each temperature parameter, weekly campylobacteriosis reports were grouped into classes of 5,000 reports forming 61 classes with one calculated incidence per class and eight different lagtypes (Fig. 2).

### Statistical methods

A four-parameter sigmoid logistic model Eq. (1) was used to describe the association between campylobacteriosis incidence and temperature. All estimated parameters were statistically significant (all p < 0.001) and robust against single values validated with the bootstrap procedure.

1 $${\text{log4}}:y = b0 + b{1}/({1} + {\exp}( - b{2} * (x - b{3})))$$

To estimate the steepest incidence increase as a function of temperature, the slope in the inflexion point (b3), has been calculated for each sigmoid function as:

2 $$slope = (b1 * b2)/4$$

A segmented linear regression has been performed for the most significant temperature parameters to identify thresholds and examine the relationship between campylobacteriosis incidence and ambient temperature at relevant intervals across the temperature range^36^.

Statistical analysis was carried out using STATA (Version IC 13.1) and Mathematica (Version 11.3). For all regression analysis, the median of each class of 5,000 reports was used because of its robustness against extreme values. To identify the most significant temperature parameter and lagtype combination, the 5% best outcomes of the sum of squared deviation from the averaged incidence (in the following called ‘SSDA’) and R-square of the regression model were selected for each lag-temperature combination. SSDA defines the deviation of incidence outcome in the respective week from the overall averaged incidence per temperature parameter and lagtype. The deviation from the averaged incidence is considered to quantify the influence of the tested temperature parameter on the incidence outcome. Thus, the higher the SSDA value, the better the incidence outcome is explained by the tested temperature parameter. The averaged incidence is calculated by summing up all reported incidence values and dividing it by the total number of incidence entries:

3 $${\text{expected incidence}} = \sum_{{\left( {k = 1} \right)}}^{N i\left( k \right)/N}$$

It is assumed that the averaged incidence is the resulting incidence value if there is no association between campylobacteriosis incidence and ambient temperature. To examine temporal patterns in the association between ambient temperature and incidence, the results of R-square and SSDA were plotted, and lagtypes per temperature parameter were linked to show a temporal evolution of the association. For regression analysis, the two extreme classes, class 1 and 61, were omitted for several reasons. Firstly, the extreme upper class of each temperature parameter contains unequal class entries compared to the other classes containing 5,000 reports as predefined by the method. Unequal classes may lead to skewed results. Secondly, the class widths of class 1 and 61 are much broader than those of all other classes, which causes difficulties when comparing classes. In addition, extreme classes defined by the weather conditions overrepresent a few regions of extreme weather conditions and as a result, are not representative for all reporting areas for the whole of Germany.

## Results

A statistically significant association between local ambient temperature and local campylobacteriosis incidence in Germany was found. All sigmoid regressions for each temperature parameter and lagtype explained more than 97% of the variation in incidence outcome. R-square ranged from 0.9715 to 0.9959 ($\overline{x} = { }0.99$). The SSDA ranged from 7.9291 to 12.4871 (*x̅* = 10.8995) for all temperature parameters and each lagtype.

The steepest gradient for all temperature parameters averaged 0.1 ($SD\left(m\right)=0.01).$ Thus, the sigmoid regression estimated that a 5 °C increase in temperature is associated with an incidence increase of 0.52 new cases per 100,000 inhabitants per week.

**Figure 3:**
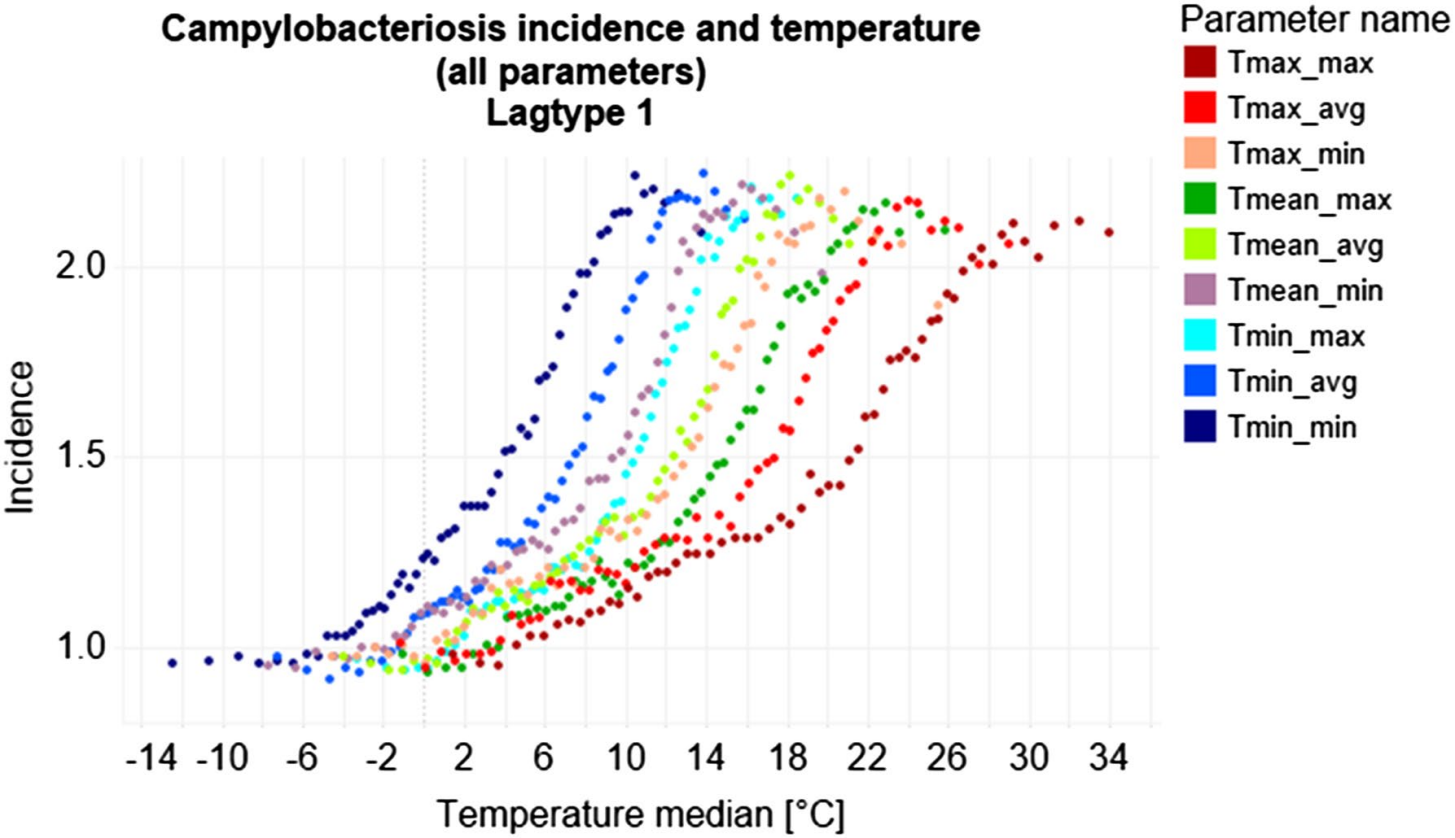
Scatterplot of all temperature parameters in lagtype 1. This graph was produced using the dashboard function of Tableau Desktop (Version 2019.4.0, URL: https://www.tableau.com/de-de/support/releases/desktop/2019.4).

**Figure 4:**
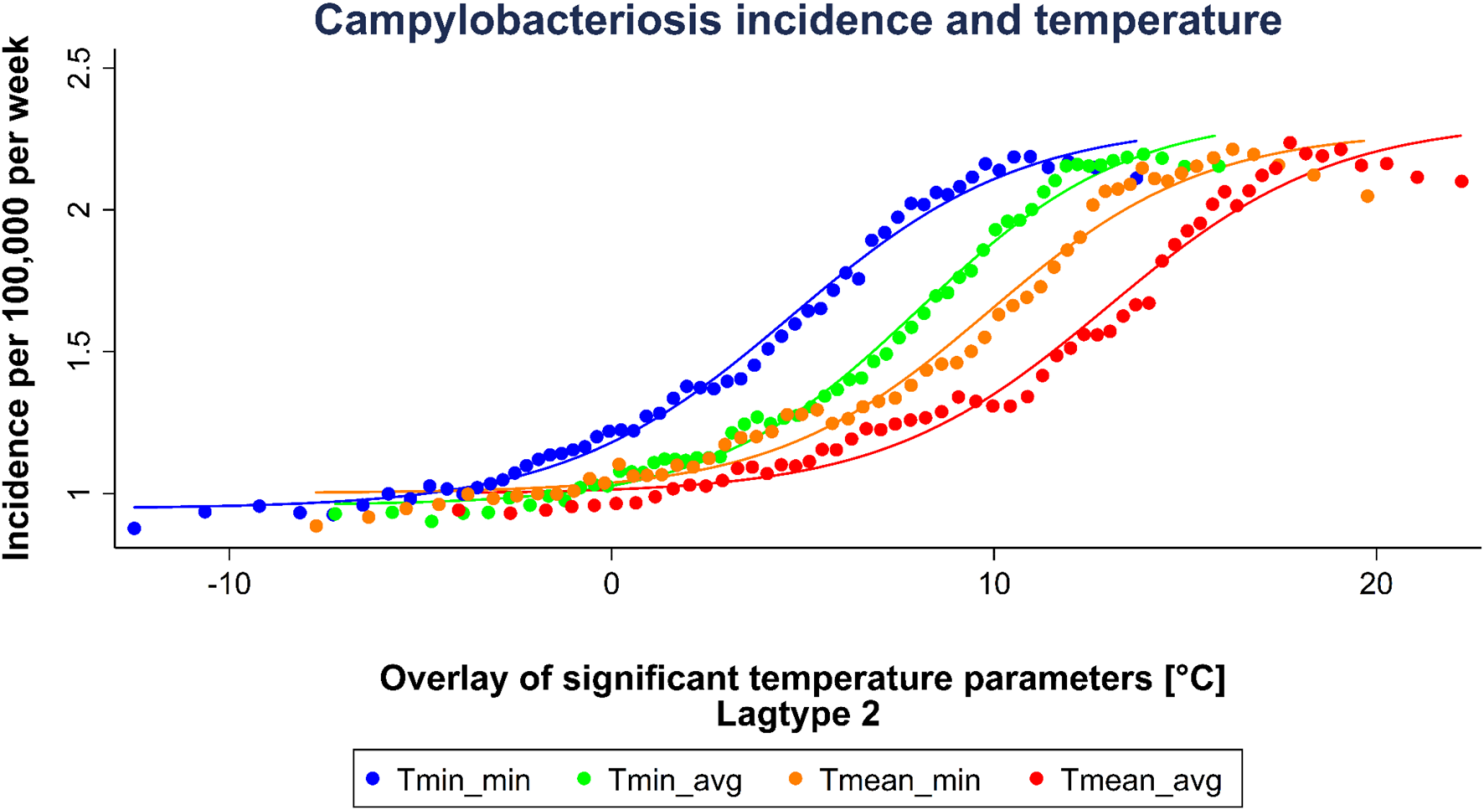
Sigmoid regression of significant temperature parameters selected by maximum SSDA values in lagtype 2. ‘Tmin_min’ represents the weekly minimum of daily minimum temperature, ‘Tmin_avg’ the weekly average of daily minimum temperature, ‘Tmean_min’ the weekly minimum of daily mean temperature, and Tmean_avg’ the weekly average of daily mean temperature. This graph was generated using STATA (Version IC 13.1, URL: https://www.stata.com/products/).

Within a temperature corridor of − 5 °C to 28 °C, incidence increased with rising ambient temperature. Below and above this corridor, incidence did not significantly alter with changing temperature (Fig. 3). Among all temperature parameters, the temperature parameter with the absolute maximum incidence of 2.25 has been reached at the weekly average of daily minimum temperature (Tmin_avg) in lagtype 1 with a median temperature of 13.91 °C and a temperature range of − 7.2 °C to 15.9 °C. Most incidence changes occurred between 0 to 12 °C (Fig. 3). Highest SSDA values were reached for minimum and mean temperatures in lagtype 2 (Fig. 4, Table 1).

**Table 1:** Temperature parameters with maximum SSDA values in lagtype 2.

Temperature parameter	Incidence^max^	Temperature^median^ [°C]	Temperature range [°C]	SSDA
Tmin_avg	2.20	13.91	− 7.23–15.88	12.4871
Tmean_min	2.21	16.24	− 7.73–19.77	12.2628
Tmin_min	2.19	10.95	− 12.49–13.73	12.2021
Tmean_avg	2.24	17.74	− 4.00–22.23	12.1615

This table was created using Microsoft Office 365 ProPlus (Version 2001, URL: https://www.microsoft.com/de-de/microsoft-365).

**Figure 5:**
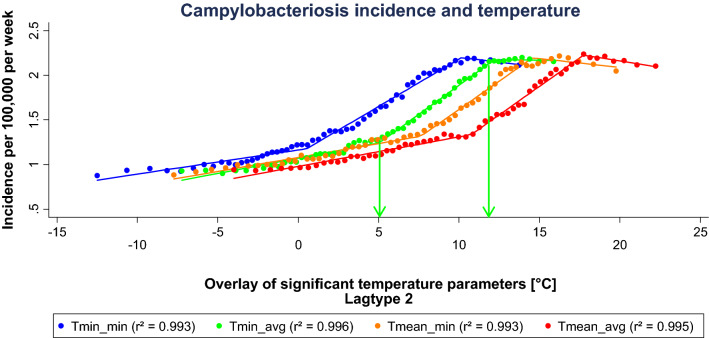
Segmented linear regression of significant minimum and mean temperature parameters selected by maximum SSDA values in lagtype 2. The arrows mark the lower and upper threshold for ‘Tmin_avg’ as example. This graph was generated using STATA (Version IC 13.1, URL: https://www.stata.com/products/).

**Figure 6:**
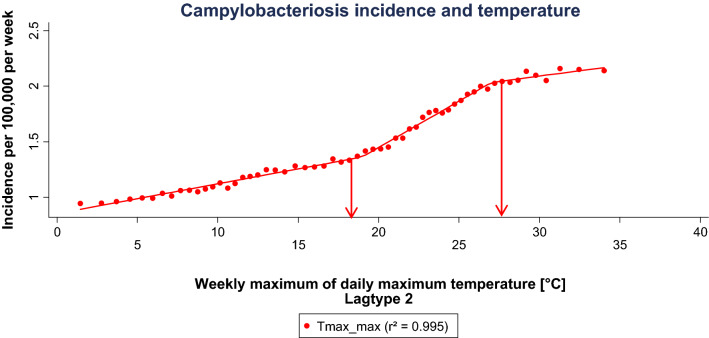
Segmented linear regression of the weekly maximum of daily maximum temperature in lagtype 2. The arrows mark the lower and upper threshold. This graph was generated using STATA (Version IC 13.1, URL: https://www.stata.com/products/).

The extended evaluation of selected parameters showed different thresholds across the segmented regression (Figs. 5, 6, Table 2). Among the temperature parameters in lagtype 2, the lowest threshold was reached at 0.53 °C mean temperature, whereas the highest upper threshold is reached at a mean temperature of 27.02 °C. The inclination of the first regression segment of each parameter shows a very shallow increase of 0.03. From the lower to the upper threshold, a steeper increase of 0.08 to 0.13 can be observed. From the upper threshold, a slightly negative to a shallow positive inclination of  − 0.02 to 0.02 is reached. As an example, at the weekly average of daily minimum temperature be tween 5.1 to 12.2 °C, the steepest incidence increase can be observed with 0.13 per 1 °C temperature increase. Above the upper threshold incidence gradually declines.

**Table 2:** Results of the segmented linear regression of selected temperature parameters in lagtype 2.

	Lower threshold [°C]	Upper threshold [°C]		Inclination (1)		Inclination (2)		Inclination (3)
Tmin_min	0.53	10.15		0.03		0.11		− 0.02
Tmin_avg	5.07	12.20		0.03		0.13		− 0.002
Tmean_min	7.63	14.57		0.03		0.13		− 0.02
Tmean_avg	10.76	17.74		0.03		0.13		− 0.03
Tmax_max	18.96	27.02		0.03		0.08		0.02

This table was created using Microsoft Office 365 ProPlus (Version 2001, URL: https://www.microsoft.com/de-de/microsoft-365).

**Figure 7:**
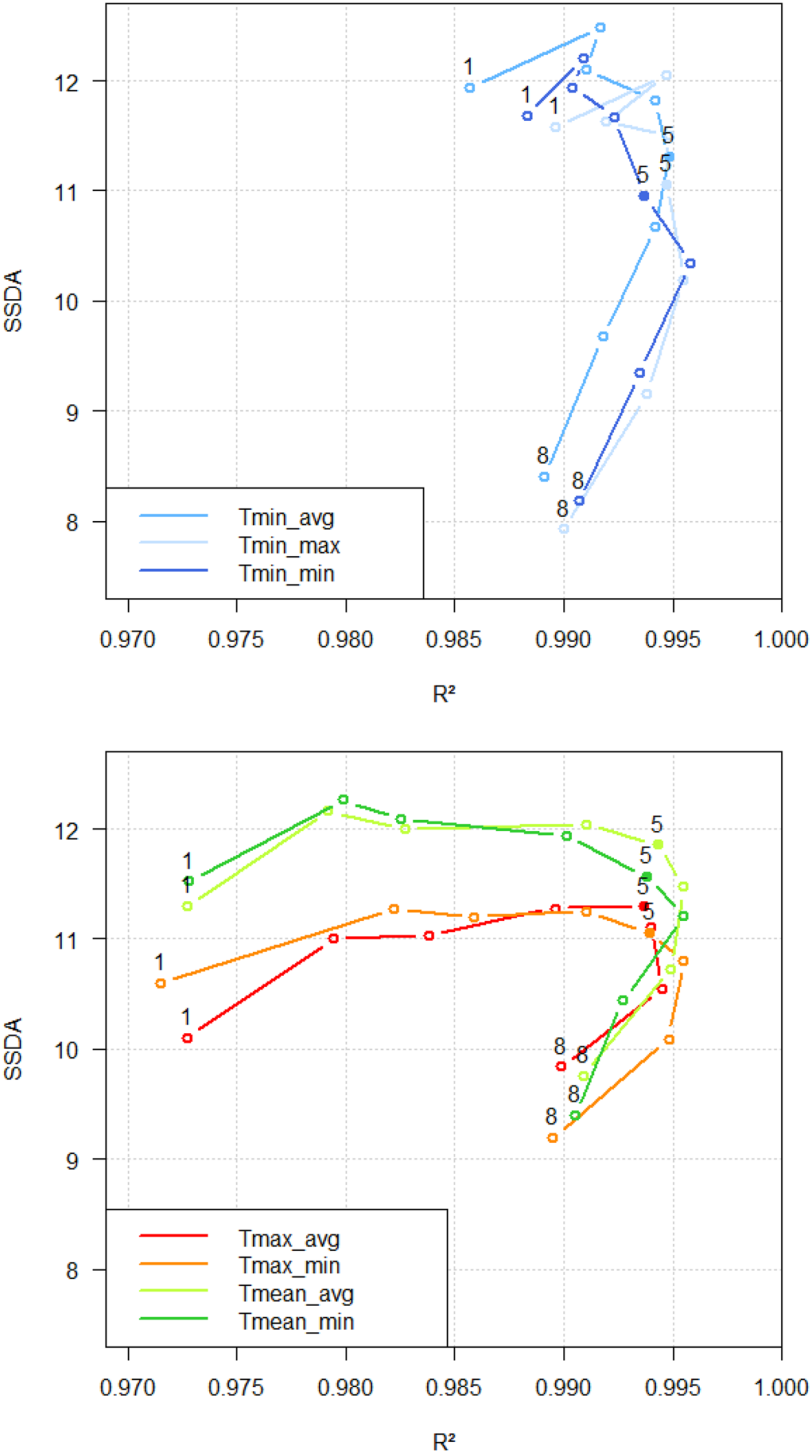
Lagtype pattern and temporal evolution. Number 1 equals Lagtype 1, and number 8 equals Lagtype 8, for example. Top chart: Weekly minimum temperature parameters. Lower chart: Lower weekly mean and maximum temperature parameters. These graphs were plotted using RStudio (Version 1.0.153, URL: https://rstudio.com/products/rstudio/older-versions/).

The correlation via plotted R-square and SSDA values showed an evolution along lagtype 1 to 8 for all temperature parameters (Fig. 7). The correlation was maximal in lagtype 4 and 5 at weekly mean and maximum temperature parameters, and for weekly minimum temperature parameters, an offset of two weeks was most significant.

## Discussion

The small-scaled approach found robust evidence of a positive correlation between local campylobacteriosis incidence and local temperature in Germany and can reproduce the correlation found at low resolutions in previous studies^26,27,31^. The strength of the association and the identification of significant lagtypes provides evidence for the influence of ambient temperature on campylobacteriosis. The described temperature corridor where incidence alters with changing temperature has been observed in previous studies^31^.

The parallelism of the segmented linear regressions and inclinations across the whole observed temperature range underlines the described association between campylobacteriosis and ambient temperature and underpins the robustness of the model. At the weekly average of daily minimum temperature from 5.1 to 12.2 °C, the steepest increase of incidence per temperature increase is apparent. In the context of climate change, days with a warmer minimum, mean and maximum temperature will become more frequent in Germany. Therefore, an increased temperature effect on campylobacteriosis incidence can be expected in the future.

The identified upper-temperature threshold with a maximum incidence of approximately 2.2 among all temperature parameters could be explained by non-climatic parameters which stabilise incidence at this level. At the upper threshold, the influential factors resulting in the observed incidence have unfolded their full temperature-associated infectious potential. Stabilising factors of campylobacteriosis incidence may be hygiene standards in food production, public awareness, and the socio-economic precondition of adequate food handling as well as diet habits specific for Germany.

The comparable correlation over the whole observed temperature range, as well as the identified temperature corridor, underline campylobacters’ ability to survive over a wide range of environmental conditions^19,22^. The observed maximal association to incidence at minimum and mean temperatures suggests that these temperature parameters are most causative for increasing campylobacteriosis incidence. Our results are consistent with other studies’ findings^22,37^.

The ability of campylobacters to survive over a wide range of temperatures is a precondition of human infection. However, the positive correlation between incidence and temperature cannot be explained by the microbiology of campylobacters solely. Increasing temperatures, especially between 5 and 25 °C, lead to increased inactivation of *Campylobacter *in vitro, while their survival is prolonged at cold temperatures^19,22,37^. These findings are contradictory to the observed relationship of our study. However, it can explain the higher incidence at average minimum temperatures.

The discrepancy between *Campylobacter* microbiology and campylobacteriosis incidence in increasing temperature conditions remains and leads to the conclusion that the weather-sensitive mechanisms on campylobacteriosis must lie outside the direct weather-pathogen relationship. These factors, themselves temperature-sensitive, are assumed to be the driving force in human campylobacteriosis at increasing temperature, outweighing the direct relationship between pathogen and temperature when the whole chain of infection is considered.

In addition to temperature, other climatic factors have been related to campylobacteriosis, such as precipitation and UV-radiation. Inconsistent evidence exists on the association between precipitation and campylobacteriosis incidence. Some studies found conflicting results or no significant association between campylobacteriosis and precipitation^23,26^. Other authors suggest heavy rain events as well as very low rainfall of 20 mm and below per week to be associated with an increased risk of campylobacteriosis, especially in campylobacteriosis outbreaks due to contaminated drinking water^38^. Rechenburg and Kistemann showed that elevated bacterial river contamination coincides with heavy rain events in summer^39^. These contaminated surface waters may become a health risk when used for recreational purposes or irrigation of cultivated plants^39^.

**Figure 8:**
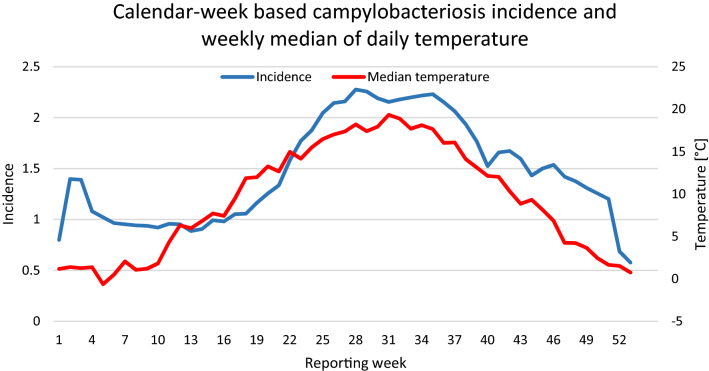
Campylobacteriosis incidence per reporting week and median temperature, averaged from 2001 until 2014 in Germany. This graph was created using Microsoft Office 365 ProPlus (Version 2001, URL: https://www.microsoft.com/de-de/microsoft-365).

An extended examination of precipitation parameters has been performed in the underlying dissertation of this paper^Supplementary information,40^. It gives evidence for a strong positive correlation between local precipitation and campylobacteriosis incidence in Germany. It was found that the steep incidence increase in week 17 to 28 is paralleling the steep increase of the weekly median of daily maximum precipitation. This may in part explain the asymmetric nature of the *Campylobacter* seasonality seen in Fig. 8.

Increased UV-radiation and coinciding higher temperatures were associated with a decline of campylobacters in river water during the summer months^41^. Other studies found a significant association with *Campylobacter* rates but best performing models where those that included temperature as cofounding variable^26^.

Despite climatic factors, various non-climatic temperature-sensitive drivers of human campylobacteriosis have been suggested. *Campylobacter* spp. is more prevalent from spring to autumn in food-producing animals such as poultry, when ambient temperatures show a seasonal increase^23^. However, findings from several studies indicate that the seasonal increase of campylobacters in chicken is not always preceding the peak of human campylobacteriosis, in some cases coinciding, or not peaking at all^24,27^. The seasonal rise of human campylobacteriosis has been linked with a higher prevalence of vectors, such as flies that carry infected material and may contaminate broiler flocks^42,43^. Several studies provide evidence for a reduced prevalence of *Campylobacter* spp. in broiler chicken after fly screens had been installed in broiler houses^44–46^. This linkage especially applies in mixed farms where cows are housed close to broiler stalls^47^. Nichols et al. related the development time of *M. domestica* larvae to seasonal patterns of temperature to underline a relationship between human campylobacteriosis and ambient temperature^43^. This hypothesis is backed by the fact that the seasonal campylobacteriosis incidence pattern echoes the annual fly population of a steep increase in spring and a slow decline in autumn. In contrast, the temperature profile in spring is the mirrored profile of autumn^43^. An explanation for the asymmetry is provided by the authors Patrick et al. (2004), who suggest that fly activity and breeding require several consecutive warm days to complete a life cycle^48^. Therefore, sustained high temperatures in summer could explain higher flocks contamination in autumn compared to spring and as a consequence, higher human campylobacteriosis incidences. As a result, rather than intermittent heat days, a consistency of warm temperatures is critical for predicting campylobacteriosis infection in broiler flocks and humans. However, the national application of fly nets in broiler houses as suggested by the European Commission in 2018^47^, has not been established systematically yet. Therefore, the relative impact on human campylobacteriosis is uncertain to-date and an avenue for further investigations.

Apart from vector prevalence, human behaviour is assumed to be highly weather-sensitive^48^, including outdoor recreational activities such as swimming in natural lakes and food culture like barbecuing. Agricultural activities, such as grazing farm animals or manure spread, also show temperature dependencies with a seasonal increase from spring throughout autumn^49^. Manure contains high amounts of zoonotic pathogens, including *Campylobacter spp.* The spread of manure is subject to firm regulations in Germany. It is restricted between the harvest of the last main crop, mainly barley and wheat, until the end of January^50^. These human behaviour-led risk factors may help explain part of the incidence summer peak^7^. A second smaller incidence peak in early January has been observed in several European countries and other foodborne bacterial diseases (Fig. 8)^3,51^. The winter peak cannot be explained by warm temperatures but has been attributed to an increased *Campylobacter*-contaminated food- and travel-related exposure during the festive season at the end of the year. This linkage underlines the relevance of human (diet) behaviour for foodborne diseases such as campylobacteriosis^25,52^. It should be pointed out, that reduced access to health care facilities during public holidays in the last week of December probably accounts for a reporting delay artefact in the winter incidence peak^25^. In the light of the weather dependencies of human behaviour, the identified temperature parameters are assumed to act as prognostic indicators for high-risk human behaviour leading to a more frequent exposure to campylobacters, which is followed by an increased incidence. Although the role of human behaviour is complex and underlies fluctuations, it might be in part explanatory for our study’s findings. However, the weather-sensitive mechanism driving campylobacteriosis incidence is yet to be identified. Elucidation is needed at what stages of the chain of infection temperature is directly linked to campylobacteriosis. The harmonisation of *Campylobacter* monitoring in Europe is a prerequisite to deconstruct trends and sources of campylobacteriosis along the food chain^3^. As a first-order approximation, the lengths of significant lagtypes can help to highlight stages in the infection pathway where the temperature may be most influential. The identified time lags offer a time window for campylobacteriosis forecast and may help epidemiologists and health authorities to improve campylobacteriosis local early warning systems.

It was demonstrated that incidence strongly associates with the average minimum temperatures two weeks before. The results indicate that average minimum temperatures are especially relevant in the recent part of the chain of infection. Along the food chain, this period includes slaughter, transport, food storage, processing, and consumption. A short delay provides evidence for a possible causal relationship between local ambient temperature and campylobacteriosis outcome. We suggest that the identified temperature ranges encourage risky human (diet) behaviour and subsequently lead to an increase in campylobacteriosis incidence. Consequently, human (diet) behaviour in the context of foodborne diseases is a crucial target for mitigation strategies.

## Conclusion

This study demonstrates a strong association between temperature and campylobacteriosis in Germany. The influence of temperature on human campylobacteriosis is likely to be indirect and especially relevant in the recent part of the infection chain. We suggest that campylobacteriosis incidence is not the result of a single driver but the integral of multiple climatic and non-climatic factors. The asymmetric nature of *Campylobacter* seasonality underlines this conclusion and gives avenues for further investigations on potentially associated parameters. Although statements can only be derived from the observation period, an increase in campylobacteriosis incidence is likely with projected warmer temperatures in the context of climate change.

## Supplementary information

Supplementary file1

Supplementary file2

## References

[1] WHO. The global view of campylobacteriosis. Report of an expert consultation (2013).

[2] RKI. Infektionsepidemiologisches Jahrbuch 2016. Available at https://www.rki.de/DE/Content/Infekt/Jahrbuch/Jahrbuecher/2016.html?nn=2374622 (2016).

[3] ECDC. The European Union One Health 2018 Zoonoses Report. Available at https://www.ecdc.europa.eu/en/publications-data/european-union-one-health-2018-zoonoses-report (2019). 10.2903/j.efsa.2019.5926 PMC7055727 32626211

[4] Evans MR, Ribeiro CD, Salmon RL (2003). Hazards of healthy living: Bottled water and salad vegetables as risk factors for Campylobacter infection. Emerg. Infect. Dis..

[5] EFSA (2016). The European Union summary report on trends and sources of zoonoses zoonotic agents and food-borne outbreaks in 2015. EFSA J..

[6] Wolfs TF (2001). Neonatal sepsis by Campylobacter jejuni: Genetically proven transmission from a household puppy. Clin. Infect. Dis..

[7] Schonberg-Norio D (2004). Swimming and Campylobacter infections. Emerg. Infect. Dis..

[8] WHO. Campylobacter. Available at https://www.who.int/news-room/fact-sheets/detail/campylobacter (2020).

[9] BVL. Zoonosen-Monitoring 2018 (2019).

[10] Park SF (2002). The physiology of Campylobacter species and its relevance to their role as foodborne pathogens. Int. J. Food Microbiol..

[11] Vries SP (2017). Genome-wide fitness analyses of the foodborne pathogen Campylobacter jejuni in in vitro and in vivo models. Sci. Rep..

[12] Joshua GWP, Guthrie-Irons C, Karlyshev AV, Wren BW (2006). Biofilm formation in Campylobacter jejuni. Microbiology (Reading, England).

[13] Purdy D, Park SF (1994). Cloning, nucleotide sequence and characterization of a gene encoding superoxide dismutase from Campylobacter jejuni and Campylobacter coli. Microbiology (Reading, England).

[14] Purdy D, Cawthraw S, Dickinson JH, Newell DG, Park SF (1999). Generation of a superoxide dismutase (SOD)-deficient mutant of Campylobacter coli. Evidence for the significance of SOD in Campylobacter survival and colonization. Appl. Environ. Microbiol..

[15] Buswell CM (1998). Extended survival and persistence of Campylobacter spp. water and aquatic biofilms and their detection by immunofluorescent-antibody and -rRNA staining. Appl. Environ. Microbiol..

[16] Zhong X (2020). Campylobacter jejuni biofilm formation under aerobic conditions and inhibition by ZnO nanoparticles. Front. Microbiol..

[17] Parkhill J (2000). The genome sequence of the food-borne pathogen Campylobacter jejuni reveals hypervariable sequences. Nature.

[18] Ikeda N, Karlyshev AV (2012). Putative mechanisms and biological role of coccoid form formation in Campylobacter jejuni. Eur. J. Microbiol. Immunol..

[19] Chan KF, Le Tran H, Kanenaka RY, Kathariou S (2001). Survival of clinical and poultry-derived isolates of Campylobacter jejuni at a low temperature (4°C). Appl. Environ. Microbiol..

[20] Hazeleger WC (1995). Temperature-dependent membrane fatty acid and cell physiology changes in coccoid forms of Campylobacter jejuni. Appl. Environ. Microbiol..

[21] Hazeleger WC, Wouters JA, Rombouts FM, Abee T (1998). Physiological activity of Campylobacter jejuni far below the minimal growth temperature. Appl. Environ. Microbiol..

[22] Thomas C, Hill DJ, Mabey M (1999). Evaluation of the effect of temperature and nutrients on the survival of Campylobacter spp. in water microcosms. J. Appl. Microbiol..

[23] Kovats RS (2005). Climate variability and campylobacter infection. An international study. Int. J. Biometeorol..

[24] Nylen G (2002). The seasonal distribution of campylobacter infection in nine European countries and New Zealand. Epidemiol. Infect..

[25] Bless PJ, Schmutz C, Mäusezahl D (2017). The recurrent campylobacteriosis epidemic over Christmas and New Year in European countries, 2006–2014. BMC Res. Notes.

[26] Louis VR (2005). Temperature-driven campylobacter seasonality in England and Wales. Appl. Environ. Microbiol..

[27] Hartnack S, Doherr MG, Alter T, Toutounian-Mashad K, Greiner M (2009). Campylobacter monitoring in German broiler flocks: An explorative time series analysis. Zoonoses Public Health.

[28] Tam CC, Rodrigues LC, O'Brien SJ, Hajat S (2006). Temperature dependence of reported Campylobacter infection in England, 1989–1999. Epidemiol. Infect..

[29] Djennad A (2019). Seasonality and the effects of weather on Campylobacter infections. BMC Infect. Dis..

[30] Lal A, Ikeda T, French N, Baker MG, Hales S (2013). Climate variability, weather and enteric disease incidence in New Zealand: Time series analysis. PLoS ONE.

[31] Yun J (2016). Association between the ambient temperature and the occurrence of human Salmonella and Campylobacter infections. Sci. Rep..

[32] RKI. SurvStat@RKI 2.0. Available at https://survstat.rki.de/Content/Query/Create.aspx (2019).

[33] ECAD. Home European Climate Assessment & Dataset. Available at https://eca.knmi.nl/dailydata/predefinedseries.php (2015).

[34] BKG. Verwaltungsgebiete 1:1.000.000. Available at https://www.geodatenzentrum.de/geodaten/gdz_rahmen.gdz_div?gdz_spr=deu&gdz_akt_zeile=5&gdz_anz_zeile=1&gdz_unt_zeile=16&gdz_user_id=0 (2015).

[35] Senatsverwaltung für Stadtentwicklung und Umwelt Berlin. File: Bezirke von Berlin. Available at https://www.stadtentwicklung.berlin.de/geoinformation/geodateninfrastruktur/de/geodienste/wfs.shtml (2015).

[36] Mitchell MN (2012). Interpreting and Visualizing Regression Models using Stata.

[37] Membré J-M, Laroche M, Magras C (2013). Meta-analysis of Campylobacter spp. survival data within a temperature range of 0 to 42 °C. J. Food Protect..

[38] Nichols G, Lane C, Asgari N, Verlander NQ, Charlett A (2009). Rainfall and outbreaks of drinking water related disease and in England and Wales. J. Water Health.

[39] Rechenburg A, Kistemann T (2009). Sewage effluent as a source of Campylobacter sp. in a surface water catchment. Int. J. Environ. Health Res..

[40] Julia Oberheim. *Weather Conditions and Campylobacteriosis In Germany.* Dissertation (Bonn, 2020).

[41] Obiri-Danso K, Paul N, Jones K (2001). The effects of UVB and temperature on the survival of natural populations and pure cultures of Campylobacter jejuni, Camp. coli, Camp. lari and urease-positive thermophilic campylobacters (UPTC) in surface waters. J. Appl. Microbiol..

[42] Hald B (2004). Flies and Campylobacter infection of broiler flocks. Emerg. Infect. Dis..

[43] Nichols GL (2005). Fly transmission of Campylobacter. Emerg. Infect. Dis..

[44] Hald B, Skovgård H, Pedersen K, Bunkenborg H (2008). Influxed insects as vectors for Campylobacter jejuni and Campylobacter coli in Danish broiler houses. Poult. Sci..

[45] Frosth S, Karlsson-Lindsjö O, Niazi A, Fernström L-L, Hansson I (2020). Identification of transmission routes of campylobacter and on-farm measures to reduce campylobacter in chicken. Pathogens.

[46] Bahrndorff S, Rangstrup-Christensen L, Nordentoft S, Hald B (2013). Foodborne disease prevention and broiler chickens with reduced Campylobacter infection. Emerg. Infect. Dis..

[47] DG Health and Food Safety. Mitigation measures in place for Campylobacter spp. in poultry - Publications Office of the EU. Available at https://op.europa.eu/en/publication-detail/-/publication/54ce4034-f5b8-11e7-b8f5-01aa75ed71a1/language-en/format-PDF (2018).

[48] Patrick ME (2004). Effects of climate on incidence of Campylobacter spp. in humans and prevalence in broiler flocks in Denmark. Appl. Environ. Microbiol..

[49] Lévesque S (2013). Campylobacteriosis in urban versus rural areas. A case-case study integrated with molecular typing to validate risk factors and to attribute sources of infection. PLoS ONE.

[50] Birgit Apel. Die neue Düngeverordnung 2020 - was ändert sich? - Landwirtschaftskammer Nordrhein-Westfalen. Available at https://www.landwirtschaftskammer.de/landwirtschaft/ackerbau/duengung/duengeverordnung/duev-2020.htm#:~:text=D%C3%BCngemittel%20mit%20wesentlichem%20Gehalt%20an%20Stickstoff%20wie%20z.B.%20G%C3%BClle%2C%20G%C3%A4rreste,gilt%20eine%20Sperrfrist%20vom%2001. (2020).

[51] Schmutz C, Mäusezahl D, Jost M, Baumgartner A, Mäusezahl-Feuz M (2016). Inverse trends of Campylobacter and Salmonella in Swiss surveillance data, 1988–2013. Euro Surv..

[52] Schielke A, Rosner BM, Stark K (2014). Epidemiology of campylobacteriosis in Germany—insights from 10 years of surveillance. BMC Infect. Dis..

