# Supplementary material for: Small-scaled association between ambient temperature and campylobacteriosis incidence in Germany
Source: Sci Rep. 2020 Oct 14;10:17191. doi: 10.1038/s41598-020-73865-9 (PMC7560705; doi:10.1038/s41598-020-73865-9)
Supplement: Supplementary file 2 — Supplementary file2 [file 41598_2020_73865_MOESM2_ESM.pdf]

Supporting Online Material

Small-scaled association between ambient temperature and campylobacteriosis incidence in Germany

Julia Oberheim, Christoph Höser, Guido Lüchters, Thomas Kistemann

To whom correspondence should be addressed. E-mail: juliaschulze00@googlemail.com

This material includes:

Supplementary table 1: This table shows all results of the sum squared deviation (SSDA) for each parameter-lag-combination, including all classes.

Supplementary table 2: This table shows the calculated coefficient of determination (R^2) of a sigmoid regression for each parameter-lag-combination while two extreme classes were omitted.

Supplementary excel file 1: This file includes the processed evaluation results after aggregating the data into classes of 5,000 reports. Please find the reading guide in the excel file for further explanation. Please note that in the original study besides temperature precipitation and air pressure were also analysed.

Supplementary table 1: Results: Sum of squared deviation (SSDA) of incidence vs expected incidence of all classes.

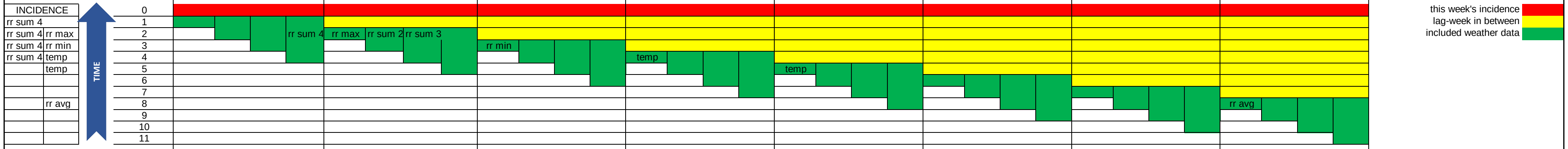

week # rel. to focus week	Lagtype 1<br>temporal offset = 1 week<br>extra lag = 0 weeks	Lagtype 2<br>temporal offset = 2 weeks<br>extra lag = 1 week	Lagtype 3<br>temporal offset = 3 weeks<br>extra lag = 2 weeks	Lagtype 4<br>temporal offset = 4 weeks<br>extra lag = 3 weeks	Lagtype 5<br>temporal offset = 5 weeks<br>extra lag = 4 weeks	Lagtype 6<br>temporal offset = 6 weeks<br>extra lag = 5 weeks	Lagtype 7<br>temporal offset = 7 weeks<br>extra lag = 6 weeks	Lagtype 8<br>temporal offset = 8 weeks<br>extra lag = 7 weeks			
**Precipitation**											
this week's daily maximum	0.7709	0.7698	0.8615	0.8634	0.8153	0.6518	0.5538	0.4022	0.8634		
this week's daily average	0.5535	0.5767	0.6271	0.6300	0.5956	0.4658	0.3714	0.2897	0.6300	0.8634	
this week's daily minimum											
**Precipitation**	1.3244	1.3465	1.4885	1.4934	1.4108	1.1176	0.9251	0.6918			1.6856
one-week sum	0.5535	0.5767	0.6271	0.6300	0.5956	0.4658	0.3714	0.2897	0.6300		
two-week sum	0.8940	0.9531	0.9763	0.9363	0.7732	0.6049	0.4666	0.2897	0.9763	1.6856	
three-week sum	1.5637	1.5833	1.6152	1.3794	1.1354	0.9025	0.7306	0.7307	1.6152		
four-week sum	1.6856	1.6545	1.5656	1.3568	1.1558	0.9195	0.6624	0.6124	1.6856		
**Precipitation week sum**	4.6967	4.7677	4.7842	4.3024	3.6599	2.8927	2.2309	1.9225			
**Temperature**											
this week's average daily maximum	10.1050	11.0032	11.0378	11.2806	11.2948	11.1080	10.5504	9.8401	11.2948	11.2948	
this week's lowest daily maximum	10.5995	11.2769	11.2007	11.2499	11.0611	10.8036	10.0934	9.1973	11.2769		
**daily mean**											
this week's maximum daily mean	10.5476	11.4433	11.5553	11.8246	11.8611	11.4247	10.6732	9.8221	11.8611		
this week's average daily mean	11.3017	12.1615	11.9977	12.0409	11.8587	11.4762	10.7292	9.7605	12.1615	12.2628	12.4871
this week's lowest daily mean	11.5280	12.2628	12.0876	11.9426	11.5686	11.2046	10.4393	9.4041	12.2628		
**daily min.**											
this week's maximum daily minimum	11.5840	12.0460	11.6275	11.5054	11.0597	10.1936	9.1510	7.9291	12.0460		
this week's average daily minimum	11.9380	12.4871	12.1030	11.8212	11.3131	10.6754	9.6742	8.4100	12.4871	12.4871	
this week's lowest daily minimum	11.6833	12.2021	11.9418	11.6724	10.9611	10.3389	9.3462	8.1833	12.2021		
**Temperature**	89.2872	94.8829	93.5514	93.3376	90.9782	87.2250	80.6570	72.5464			

Supplementary table 2: Results: Coefficient of determination (R^2) for sigmoid regression (two extreme classes omitted).

R^2 (coefficient of determination) for sigmoid regression (extreme classes omitted)

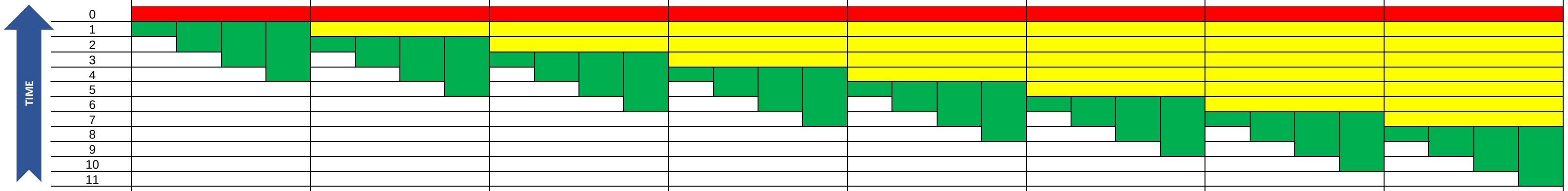

this week's incidence
lag-week in between
included weather data

week # rel. to focus week	**Lagtype 1** temporal offset = 1 week extra lag = 0 weeks	**Lagtype 2** temporal offset = 2 weeks extra lag = 1 week	**Lagtype 3** temporal offset = 3 weeks extra lag = 2 weeks	**Lagtype 4** temporal offset = 4 weeks extra lag = 3 weeks	**Lagtype 5** temporal offset = 5 weeks extra lag = 4 weeks	**Lagtype 6** temporal offset = 6 weeks extra lag = 5 weeks	**Lagtype 7** temporal offset = 7 weeks extra lag = 6 weeks	**Lagtype 8** temporal offset = 8 weeks extra lag = 7 weeks			
**Precipitation**											
this week's daily maximum	0.9396	0.9324	0.9249	0.9214	0.9178	0.8704	0.8033	0.7569	0.9396		
this week's daily average	0.9124	0.9153	0.8977	0.9467	0.9278	0.8858	0.8294	0.6379	0.9467	0.9467	
this week's daily minimum											
**Precipitation**	1.8520	1.8477	1.8226	1.8681	1.8456	1.7562	1.6327	1.3948			0.9587
one-week sum	0.9124	0.9153	0.8974	0.9467	0.9279	0.8858	0.8294	0.6379	0.9467		
two-week sum	0.9523	0.9517	0.9550	0.9402	0.9308	0.9061	0.7755	0.6690	0.9550	0.9587	
three-week sum	0.9250	0.9361	0.9256	0.8897	0.9029	0.8246	0.7017	0.5315	0.9361		
four-week sum	0.9477	0.9587	0.9356	0.9391	0.9344	0.8855	0.7479	0.4743	0.9587		
**Precipitation week sum**	3.7374	3.7618	3.7136	3.7157	3.6960	3.5020	3.0545	2.3127			
**Temperature**											
**daily max.**											
this week's maximum daily maximum	0.9832	0.9875	0.9878	0.9921	0.9948	0.9948	0.9959	0.9929	0.9959		
this week's average daily maximum	0.9727	0.9794	0.9838	0.9896	0.9937	0.9940	0.9945	0.9899	0.9945	0.9959	
this week's lowest daily maximum	0.9715	0.9822	0.9859	0.9910	0.9939	0.9955	0.9948	0.9895	0.9955		
**daily mean**											
this week's maximum daily mean	0.9873	0.9922	0.9902	0.9931	0.9939	0.9951	0.9945	0.9935	0.9951		
this week's average daily mean	0.9727	0.9792	0.9827	0.9910	0.9943	0.9955	0.9949	0.9909	0.9955	0.9955	0.9959
this week's lowest daily mean	0.9728	0.9799	0.9825	0.9901	0.9938	0.9955	0.9927	0.9905	0.9955		
**daily min.**											
this week's maximum daily minimum	0.9896	0.9947	0.9919	0.9948	0.9947	0.9955	0.9938	0.9900	0.9955		
this week's average daily minimum	0.9857	0.9917	0.9910	0.9942	0.9948	0.9942	0.9918	0.9891	0.9948	0.9958	
this week's lowest daily minimum	0.9883	0.9909	0.9904	0.9923	0.9937	0.9958	0.9935	0.9907	0.9958		
**Temperature**	8.8238	8.8777	8.8862	8.9282	8.9476	8.9559	8.9464	8.9170			
